# Enlightening and Directing Public Opinion in Regard to the Duties, Responsibilities and Requirements of Dental Practitioners

**Published:** 1867-04

**Authors:** J. M. Rhoades


THE
DENTAL REGISTER.

VOL. XXI.] APRIL, 1867. [No. 4.

### Original Communications.

### ENLIGHTENING AND DIRECTING PUBLIC OPINION IN REGARD TO THE DUTIES, RESPONSIBILITIES AND REQUIREMENTS OF DENTAL PRACTITIONERS.

BY J. M. RHOADES.

**Read before the Ohio State Dental Association.**

There is no profession in which its members are so prone to grow negligent in endeavoring to educate the people as in the science of Dentistry; thus rendering them less qualified to judge of the requirements of the Dentist than of other professional men. Thus, having but little, if any, means of comparison, they are not disposed to give to the well qualified Dentist that preference and prominence over the quack he deserves, and thus holding out a continual inducement for the ignorant and unworthy to come in competition, and successfully, too, for a time, even with the most eminent of our profession.

This is a source of continual regret and annoyance to the honorable practitioner of Dentistry.

Yet, when we view the past, and, to some extent, the present history of Dentistry, and see how studiously the people were taught to believe that Dentistry was merely a trade or occupation like that of any other mechanical business, and he whom nature had endowed with mechanism, by cultivation of the same, would make a scientific Dentist, (mechanically speaking) and that there was nothing of a healing nature pertaining thereto.

Should we then wonder at their ignorance, or be surprised that they should still cling to their prejudices, and particularly so when we find them encouraged in this by a large body of unscrupulous charlatans, who are willing to make any and every sacrifice to gratify their own base passion to the greed of gain.

To eradicate this growing evil, in my opinion, we have but to give the people as fair an opportunity to judge our qualifications as they have of those of the other professions.

This, I think, can only be done by giving them an opportunity of acquiring a sufficient knowledge of anatomy, physiology and hygiene as to know the importance of a thorough knowledge of those subjects by one who professes to be a practitioner of Dentistry. Without this degree of knowledge, how utterly impossible is it for people to judge, or even approximate thereto.

They have those of the other learned professions; why not of ours.

The clergy's public ministrations are laid open to the criticism of all. His audience are, to a large extent, competent judges of his pulpit efforts, and if he manifests ignorance, stupidity or superficiality, he forthwith falls in public estimation to the low level he deserves.

The lawyer is brought to a more rigid test. All his legal papers must be drawn up with the most scrupulous technical accuracy, or they will fail of their object. Here all great errors are open and apparent, and involve the reputation and standing of the practitioner; and likewise before the bar the lawyer is brought in open conflict with an opponent who is ever watching to pick a flaw or take advantage of his weakness or ignorance, and, perchance before a Court fully understanding the principles involved, and who indicates in his decision which party is right.

The knowledge and abilities of the Dentist in the practice of his profession cannot be tested in the same direct and open manner. The very nature of his business precludes it.

His is an inductive science, many of the truths of which are hidden in the secret recesses of life, only to be detected and demonstrated by the closest observation, of which the uninitiated have no conception.

His qualifications can only be judged by those who know how fearfully and wonderfully we are made, and are acquainted, at least somewhat, with the hidden springs of life.

It is the want of this general information on those subjects that induces even men of eminence in literature and the arts and sciences to countenance and even encourage the most ridiculous forms of quackery.

First to disabuse the public mind of the hoary errors that have reached them through years of wrong teaching, we must give them a true idea of what constitutes rational Dentistry by teaching them its basis, teaching them that all diseases are problems to be solved by deduction; that are governed by known and fixed laws, that are as incapable of variation or change as those that govern sun and moon.

That these laws and their bearings are not all known and perfectly understood as yet by the profession itself, we admit. But that there are a sufficient number of them discovered and fully established to warrant the deduction as to their true existence, we affirm, and that the great Dental body are constantly progressing in making new discoveries and new applications which the experience of every day proves, and that the Ohio State Dental Association may be a thorough advocate and co-worker in this great and good cause is the sincere wish of your humble essayist.

